# Moiré Exciton Polaron Engineering via twisted hBN

**DOI:** 10.1021/acs.nanolett.4c04996

**Published:** 2024-12-02

**Authors:** Minhyun Cho, Biswajit Datta, Kwanghee Han, Saroj B. Chand, Pratap Chandra Adak, Sichao Yu, Fengping Li, Kenji Watanabe, Takashi Taniguchi, James Hone, Jeil Jung, Gabriele Grosso, Young Duck Kim, Vinod M. Menon

**Affiliations:** † Department of Physics, Kyung Hee University, Seoul 02447, Republic of Korea; ‡ Department of Physics, City College of New York, New York, New York 10031, United States; ¶ Photonics Initiative, Advanced Science Research Center, City University of New York, New York, New York 10031, United States; § Department of Physics, University of Seoul, Seoul 02504, Republic of Korea; ∥ Research Center for Electronic and Optical Materials, National Institute for Materials Science, 1-1 Namiki, Tsukuba 305-0044, Japan; ⊥ Research Center for Materials Nanoarchitectonics, National Institute for Materials Science, 1-1 Namiki, Tsukuba 305-0044, Japan; # Department of Mechanical Engineering, Columbia University, New York, New York 10027, United States; @ Department of Smart Cities, University of Seoul, Seoul 02504, Republic of Korea; △ Physics Program, Graduate Center, City University of New York, New York, New York 10016, United States; ∇ Department of Information Display, Kyung Hee University, Seoul 02447, Republic of Korea

**Keywords:** moiré, exciton, polaron, MoSe_2_, hexagonal boron nitride, ferroelectricity

## Abstract

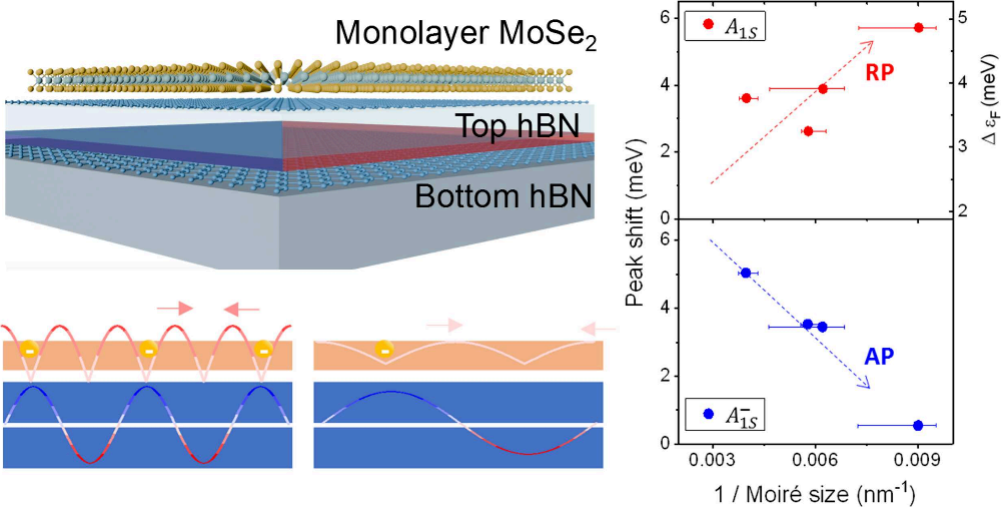

Twisted hexagonal boron nitride (thBN) exhibits ferroelectricity due to moiré superlattices with AB/BA domains. These domains possess electric dipoles, leading to a periodic electrostatic potential that can be imprinted onto other materials placed in its proximity. Here we demonstrate the remote imprinting of moiré patterns from thBN onto monolayer MoSe_2_ and investigate the changes in the exciton properties. We confirm the imprinted moiré patterns on monolayer MoSe_2_ using Kelvin probe force microscopy (KPFM) and hyperspectral photoluminescence (PL) mapping. By creating a large ferroelectric domain (∼8.7 μm), we achieve unprecedented potential modulation (∼387 ± 52 meV). We observe the formation of exciton-polarons by the ferroelectric moiré domains and investigate the optical property changes induced by the moiré pattern in monolayer MoSe_2_ by varying the moiré domain size down to ∼110 nm. Our findings highlight the potential of thBN as a platform for controlling the properties of 2D materials for optoelectronic and valleytronic applications.

A van der Waals (vdW) ferroelectric with two-dimensional (2D) crystal lattices has emerged as a promising platform for applications in next generation quantum electronic and spintronic devices.^1^ In addition to naturally occurring layered vdW ferroelectrics, there exists the intriguing possibility of achieving ferroelectricity in vdW heterostructures made of parent compounds that are not intrinsically ferroelectric.^2^ Hexagonal boron nitride (hBN) is a good example of such emergent ferroelectricity that arises from the sliding or twisting between two hBN layers.^3−6^ When two hBN layers are stacked with a slight misalignment, which is twisted hBN (thBN), the nitrogen and boron at the interface of hBN form a triangular moiré superlattice structure with AB and BA domains, exhibiting an electric dipole moment. The out of plane dipole moment has opposite directions in the AB and BA domains because of the different atomic arrangement, resulting in triangular ferroelectric moiré domains with alternating opposite polarization. Along with the ferroelectric properties, the possibility to tune the properties on demand via nanomechanical rotation^7^ or via an external electric field to induce sliding between AB and BA domains shows promise for a programmable ferroelectric platform.^8,9^

2D materials such as graphene and transition metal dichalcogenides (TMDCs) that host unique electronic and optical properties are also highly susceptible to the external environment. Therefore, there has been significant research aimed at manipulating these properties by engineering the external environment.^10−12^ Recently, studies have been conducted on imprinting moiré patterns onto different target 2D materials to investigate the effect of remote interactions.^13−16^ There have also been recent reports on imprinting moiré patterns on target 2D materials placed in the proximity of thBN resulting in localized excitons and doping.^17−19^ However, there has been no clear evidence of the imprinted moiré patterns and the modified optical properties induced by the alternating ferroelectric moiré domains on the proximitized excitons of the target material.

Here, we report on the optical properties of monolayer MoSe_2_ with imprinted moiré patterns from the underlying thBN. Moiré patterns with small twist angles ranging from 0.01 to 0.2° were created by stacking two nearly aligned hBN flakes. It was also found that twist angles below 0.01° can be realized by repeating the stacking process on the thBN. Using this approach, we achieved unprecedented potential modulation of ∼387 ± 52 meV. We confirmed the imprinting of moiré patterns on monolayer MoSe_2_ via proximity, using Kelvin probe force microscopy (KPFM) and hyperspectral PL mapping to visualize and quantify the effect. Finally, we investigated the changes in exciton properties due to the charge redistribution caused by the ferroelectric moiré domains. In TMDCs at low residual charge densities when a well-defined Fermi sea is not present, the excitons bind to the charges forming trions. At higher densities, the excitons get dressed by the Fermi sea and are called exciton-polarons, where the neutral exciton state forms the repulsive polaron (RP) and the trion state evolves into the attractive polaron (AP).^20−25^ The modulation of the charge density arising from the underlying moiré potential of the thBN results in a shift in Fermi level causing the energy shift of the exciton-polarons.

A schematic of the sample structure is shown in Figure 1a, where moiré patterns form at the interface of the two hBN layers. When the arrangement of atoms is in a parallel configuration, it results in three atomic registries: AA, AB, and BA. In this case, AB and BA configurations relax to the most stable state, forming triangular ferroelectric moiré domains with opposite polarization direction. The AB domain has a dipole moment in the downward direction, while the BA domain has a dipole moment in the upward direction. The different directions of the dipole moments induce an electric field on the surface. This in turn leads to electron redistribution in the target 2D material MoSe_2_, as shown schematically in Figure 1a. These electrons interact with the excitons to form exciton-polarons. In a previous work, it was shown that the target material placed on thBN can be affected not only by the potential difference but also by the in-plane induced electric field.^19^ The schematic of this in-plane electric field is illustrated in Figure 1b. Additionally, the direction of the in-plane electric field applied between the AB and BA domains can vary depending on whether the target material is positioned above or below the thBN. When MoSe_2_ is positioned above, electrons migrate from the AB domain to the BA domain. As a result, in Figure 1c, the Fermi level increases only in the BA domain, and in this specific domain, excitons interact with electrons to form exciton-polarons, while the AB domain contains only bare excitons. When the MoSe_2_ monolayer is positioned below the thBN, the direction of the electric field is reversed, resulting in the formation of exciton-polarons in the AB domain and bare excitons in the BA domain. The difference in Fermi level between the domains lead to different interactions between excitons and electrons, resulting in an energy difference depending on the domain. The proximity effect on MoSe_2_ can also be influenced by the difference in charge density arising from the different dipole orientations in the AB and BA domain. The energy difference between the RP and the AP can be approximated as the sum of the trion binding energy and the Fermi level as shown in Figure 1(d).^25^

**Figure 1:**
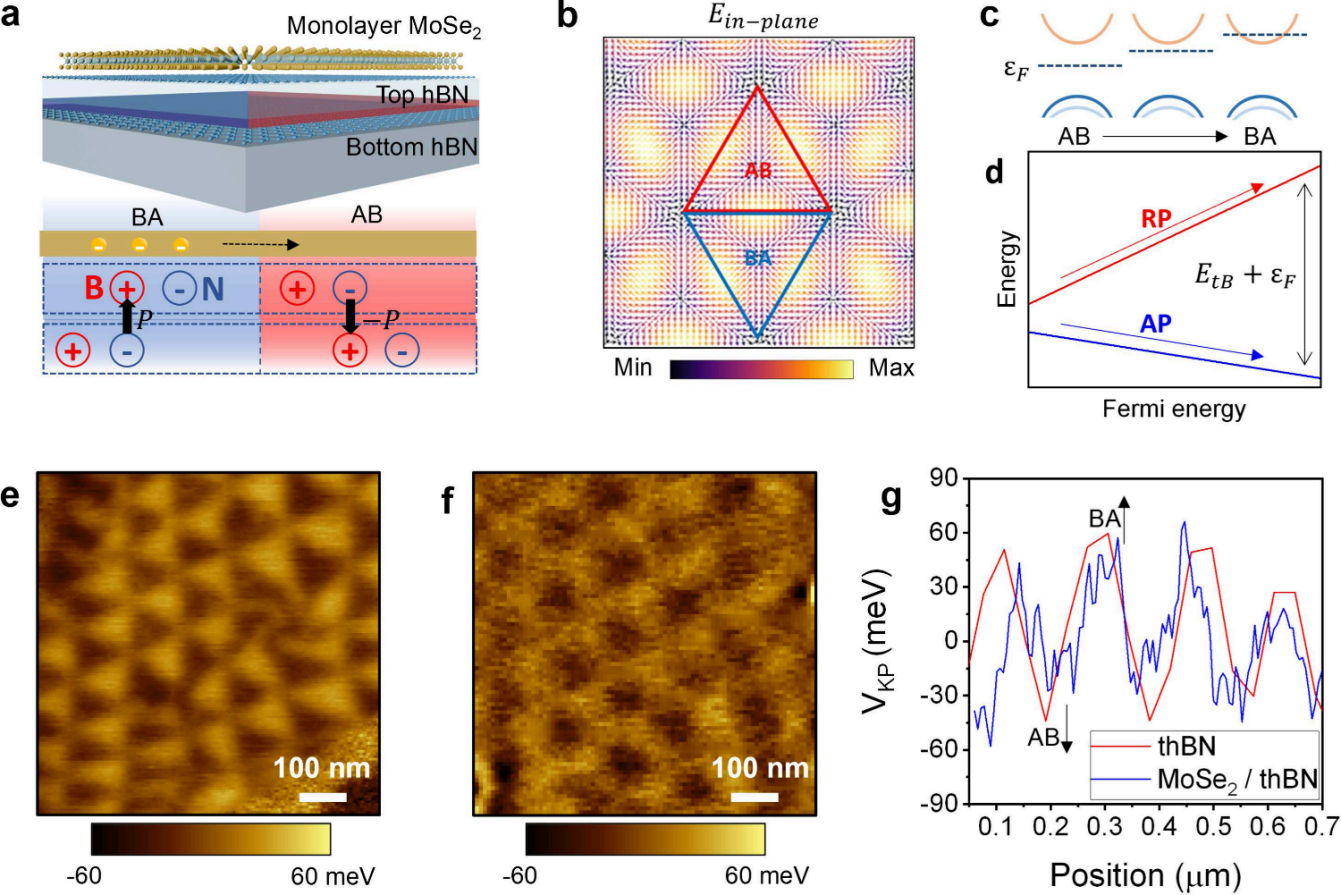
thBN moiré superlattice imprinting on the monolayer MoSe_2_. (a) Schematic of the sample. When two hBN layers are aligned in parallel, they form triangular ferroelectric moiré domains consisting of AB and BA domains at the interface. In the schematic, the lower part depicts the atomic arrangement at the boundary between the two hBN layers. Due to the relaxed atomic arrangement of boron and nitrogen, a downward dipole moment exists in the AB domain, while an upward electric dipole exists in the BA domain. Due to the electric dipole in each domain, an electric field is formed at the domain boundary. When MoSe_2_ is placed on the thBN surface, it experiences electron redistribution due to these electric fields. The excited Fermi sea in the BA domain coupled with excitons to form exciton-polarons. (b) The in-plane electric dipole schematic of thBN. When MoSe_2_ is positioned on top of thBN, the direction of the electric field is from the BA domain to the AB domain, leading to an increase in electron density and Fermi level in the BA domain. On the other hands, when MoSe_2_ is positioned below thBN, there is an increase in electron density and Fermi level in the AB domain. (c) Schematic showing the Fermi level depending on the position of MoSe_2_ on thBN domains. (d) Schematic representation of repulsive polaron (RP) and attractive polaron (AP) energies depending on Fermi level. The energy difference between two peaks increases as the Fermi level increases. (e) KPFM measurements of the moiré superlattice on thBN. The superlattice size is 161 ± 30 nm, corresponding to a twist angle around 0.089°. Top and bottom hBN thickness is 8.5 nm. (f) KPFM measurements of moiré superlattice on MoSe_2_/thBN. (g) Comparison of KPFM measurements in thBN and MoSe_2_/thBN regions. While the potential difference remains relatively constant around 110 meV, there is a noticeable increase in noise on MoSe_2_.

The fabrication of thBN involves stacking two hBN layers that are exfoliated in close location and aligned nearly perfectly in their as-exfoliated state^5^ on a SiO_2_/Si substrate using the PC (polycarbonate) dry transfer method (Figure S1).^26^ The moiré size made by this method is usually 100 nm to 1 μm, corresponding to twist angles varying from 0.0143 to 0.143°, resulting in a 100–250 meV potential difference (Figure S2). The bulk MoSe_2_ is exfoliated onto a PDMS substrate, and the monolayer MoSe_2_ is transferred directly from the PDMS substrate to the thBN. KPFM measurements are performed using the frequency modulated (FM) -KPFM method with a positive bias applied to the tip (Supporting Information). Figure 1e,f shows the KPFM images of thBN and the monolayer MoSe_2_ on the thBN, respectively. Figure S3 shows the full KPFM image of the same sample, where MoSe_2_ is placed on a part of thBN. We observe that the potential modulation in thBN is also present in MoSe_2_ with the same moiré size and magnitude. While the overall work function is lower in MoSe_2_, we observe a consistent moiré potential, confirming the presence of the proximitized moiré potential in the target materials. It exhibits moiré sizes about 161 ± 30 nm with the monolayer MoSe_2_ showing higher noise because of the charge redistribution in the MoSe_2_. Figure 1g shows line cuts of the KPFM image for the two scenarios indicating excellent agreement in both the induced potential difference around 110 meV and the moiré size. This indicates that the moiré patterns did not change during the transfer process and that the moiré potential is imprinted on the proximitized MoSe_2_. Subsequently, Raman spectroscopy was conducted to investigate any strain effects arising from the underlying moiré structure (Figure S3). The peak energies of both A_1g_ and E_2g_^1^ modes showed no change. This confirmed that no additional strain was introduced to MoSe_2_ by the underlying moiré superlattice.

The effects on the exciton properties of such small domain size (161 ± 30 nm) cannot be investigated using standard far-field spectroscopic methods, as the effects are averaged over several domains within the diffraction limited spot (Figure S3). Although in theory the size of the moiré pattern formed by two parallel hBN layers can become infinitely large, achieving a large moiré pattern has not been possible thus far when artificially aligning two hBN layers. To realize domains that can be investigated using far field spectroscopic measurements, we developed a technique to realize large domain sizes ranging from 1 to 8.7 μm. This technique relies on the tendency of the thBN to consistently relax to the most stable state of 0° when the stacking process is repeated. See section 1 in the Supporting Information for details on the repeated transfer process to realize large domains. Figure 2a shows the KPFM image of the sample with the largest moiré size among the stacked devices. To check for additional relaxation through heating, we annealed the sample and then conducted KPFM measurements (Figure S4). The moiré size was found to remain unchanged after annealing. When the second transfer process was conducted on the same substrate, as shown in Figure 2b, the moiré size increased to a maximum of 8.7 μm. The angle moved closer to 0° only after we repeated the transfer step.

**Figure 2:**
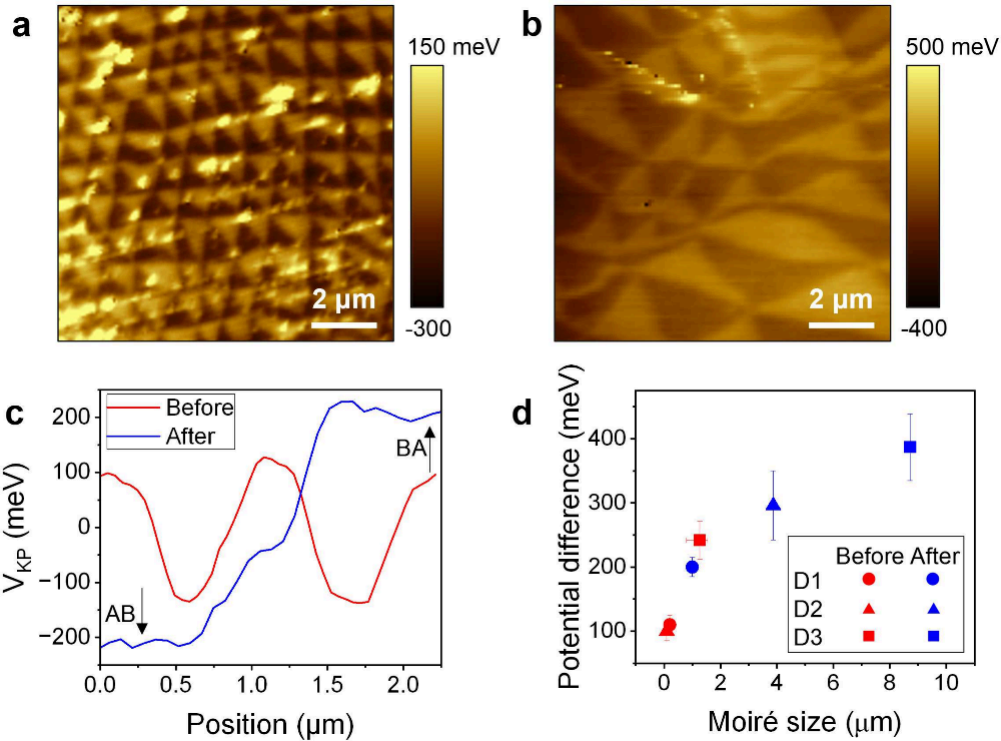
Moiré potential maximization after repeating the transfer process. (a) KPFM image measured before repeating the transfer of thBN. The moiré size ranges from 793 to 1266 nm, corresponding to twist angles between 0.011 and 0.019°. The top hBN thickness is 7 nm, and the bottom hBN thickness is 18 nm. (b) KPFM image measured after repeating the transfer process on the same SiO_2_/Si substrate. The moiré size increased significantly, ranging from approximately 1310 to 8720 nm, an increase of over 10 times. The angle in this region is below 0.01°. (c) Change in moiré potential before and after repeating transfer process in (a) and (b). Before repeating the transfer process, the potential is 240 ± 30 meV (red line), and after repeating the transfer process, it increases to 387 ± 52 meV (blue line), along with an increase in moiré size. (d) Potential difference versus moiré size for device 1 (circle), device 2 (triangle), and device 3 (rectangle). After repeating the transfer process, we marked the maximum moiré size values among the various sizes observed.

This relaxation to 0° is consistent with studies of bulk BN microrotators, where 0 and 60° configurations are the most stable.^7^ In this range of aligned angles, rigid moiré patterns relaxed into specific shapes. PFM studies of these patterns reveal strain accumulation at domain boundaries.^5,27^ In the second stacking process, the two hBN flakes relax into large AB and BA domains with minimal domain boundaries, thus reaching their most stable configuration. In Figure 2c, we compare the potential difference before and after repeating the transfer process. Following the second transfer, the potential difference exhibits a much larger value of 387 ± 52 meV compared to the initially formed potential difference of 242 ± 30 meV.

As the moiré size increases, the area ratio of stable AB and BA domains to unstable domains increases,^28^ and the Coulomb interaction between moiré domains decreases. Consequently, the observed moiré potential is much larger than in previous reports.^18^ In Figure 2d, we show the maximum potential difference for the three devices after the first and second transfers. After the second transfer, the size of the domain increases, resulting in a range of moiré sizes with the maximum moiré size indicated. The potential before and after transfer in Figure 2c corresponds to device 3. Similar KPFM results for other devices are listed in Figure S4. The magnitude of the moiré potential is proportional to the strength of the dipole moment in the thBN, according to the equation:^17^
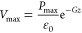
where *V*_max_ is the maximum potential difference between AB and BA domains, *P*_max_ is the maximum interlayer charge polarization, ε_0_ is the vacuum permittivity, *G* is , where *a* is moiré period size, and *z* is the distance from the target material to the interface of two hBN layers. The previously observed maximum potential difference in single thBN was ∼203 meV when the *P*_max_ value is around 2.01 pC/m.^18^ This value agrees well when the moiré size is below 1 μm. In contrast, following the second transfer, the moiré size increases with additional relaxation and the maximum potential difference we observe is ∼387 ± 52 meV, which is 1.91 times larger than previous results.

The realization of larger moiré domains allowed us to carry out hyperspectral imaging of the exciton luminescence (see the Supporting Information). Figure 3a shows the results of these measurements where thBN underwent a second transfer onto MoSe_2_/hBN to create large moiré domains, allowing us to spectroscopically investigate the exciton properties in the different moiré domains. We observe distinct PL emisison pattern from the AB and BA domains. In Figure 3(b), we show the intensity ratio where the A_1S_^–^ peak intensity is normalized to the A_1S_ peak intensity. Furthermore, the hyperspectral imaging clearly demonstrates the moiré patterns from the intensity, energy, and line width of two states (A_1S_^–^, A_1S_) in the far field (Figure S5). Figure 3c shows the contour plot of PL spectra along the white arrow in Figure 3(b). The line cuts at the AB and BA domains and the domain boundary are shown in Figure 3(d). The peak shifts of two states (A_1S_^–^, A_1S_) as a function of position are shown in Figure 3(e) clearly indicating the opposite trends for the AP and RP states. The A_1S_ peak shows a blue shift of ∼6 meV, and the A_1S_^–^ peak shows a red shift of ∼1 meV. The difference in Fermi levels between the domains results in distinct interactions between excitons and electrons, leading to domain-dependent energy shifts. In MoSe_2_, both exciton and trion states exist, and their energy differences arise from repulsive and attractive interactions with electrons, forming the RP and the AP, respectively. The energy difference between the two states observed in Figure 3e is shown in Figure 3f. Based on this energy difference, we determined the Fermi level in each domain using the equation^25^
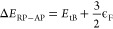
where *E*_tB_ is the trion binding energy and Δ*E*_RP–AP_ is the energy splitting between the RP and the AP. The relationship between the Fermi level, ϵ_F_, and the energy difference of the two peaks was obtained through linear fitting. The Fermi level increases along the domain with maximum increase in Fermi level estimated to be ∼4.54 meV across the boundary. The trion binding energy has a value of 20.85 meV. Here, we highlight that the peak shift is mostly due to the RP. When MoSe_2_ is positioned below the thBN, the direction of the in-plane electric dipole points from the AB to the BA domain. The increased Fermi level in the AB domain induces the formation of exciton-polarons exclusively in this domain. Conversely, the excitons in the BA domain, where the Fermi level is reduced, approaches the neutral exciton state.

**Figure 3:**
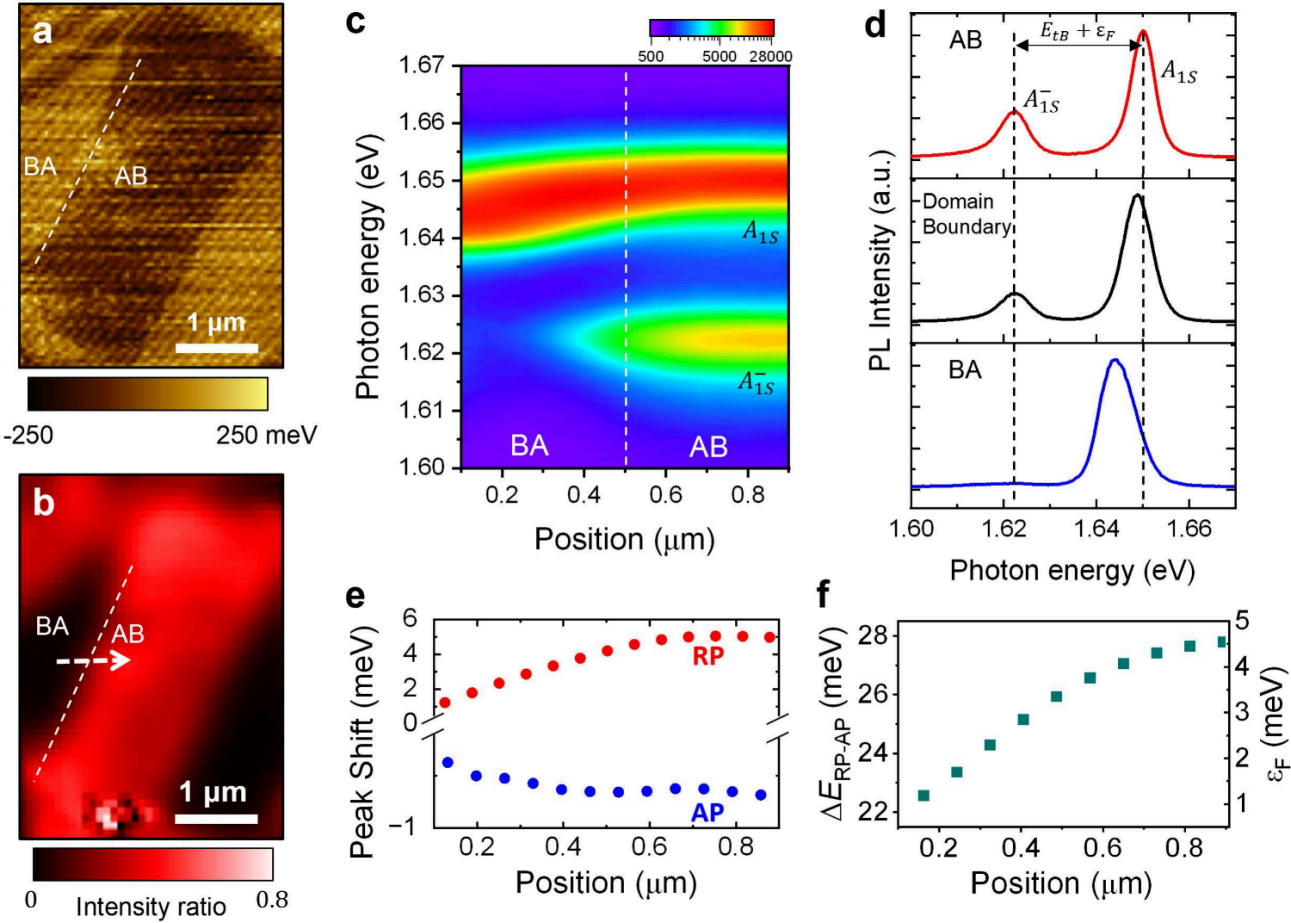
Hyperspectral imaging of exciton-polaron in thBN/MoSe_2_/hBN sample. (a) KPFM measurements of thBN/MoSe_2_/hBN sample. Twisted hBN consists of 5 nm top hBN and 5 nm bottom hBN. (b) Hyperspectral PL imaging with a 2.33 eV CW laser excitation at the same location as (a). The measured temperature is 8 K. The A_1S_^–^ peak intensity is normalized by A_1S_ peak intensity. A high intensity ratio is observed in the AB domain, while a value close to 0 is observed in the BA domain, allowing the moiré pattern to be clearly visualized. (c) Contour plot of PL spectra along the white arrow in (b). (d) Normalized PL spectra of A_1S_^–^, A_1S_ peak in AB, BA domains and near the domain boundary. The A_1S_^–^ peak position shifts from 1.623 eV in the BA domain to 1.622 eV in the AB domain. The A_1S_ peak position shifts from 1.644 eV in the BA domain to 1.650 eV in the AB domain. (e) Peak shift as a function of position. The A_1S_^–^ peak shows a red shift corresponding to an attractive polaron. The A_1S_ peak shows a blue shift corresponding to a repulsive polaron. (f) Energy splitting(Δ*E*_RP–AP_) between the repulsive polaron and the attractive polaron and the Fermi level (ϵ_F_) corresponding to this energy difference.

In addition to the 1s excitons, the excited states of excitons (Rydberg) are also affected by the underlying moiré superlattice. Figure 4a shows a hyperspectral PL image of the A_2S_ Rydberg exciton intensity, revealing a distinct spatial distribution across the AB and BA domains. The photon energy along the white dotted arrow in Figure 4a is plotted as a contour map in Figure 4b, along with line cuts in the two domains showing the PL spectra in Figure 4c. Similar to the A_1S_ exciton, the A_2S_ exciton also shifts in energy from 1.790 to 1.799 eV. Figure 4d shows the peak shift of the Rydberg exciton-polaron (A_2S_) which is observed to be larger than that of the ground state exciton-polaron (A_1S_). This observation indicates that the higher energy Rydberg states are more sensitive to the moiré potential and exhibit stronger exciton–electron interaction compared to the ground state. This behavior is consistent with previous studies on Rydberg exciton-polarons.^13,29^

**Figure 4:**
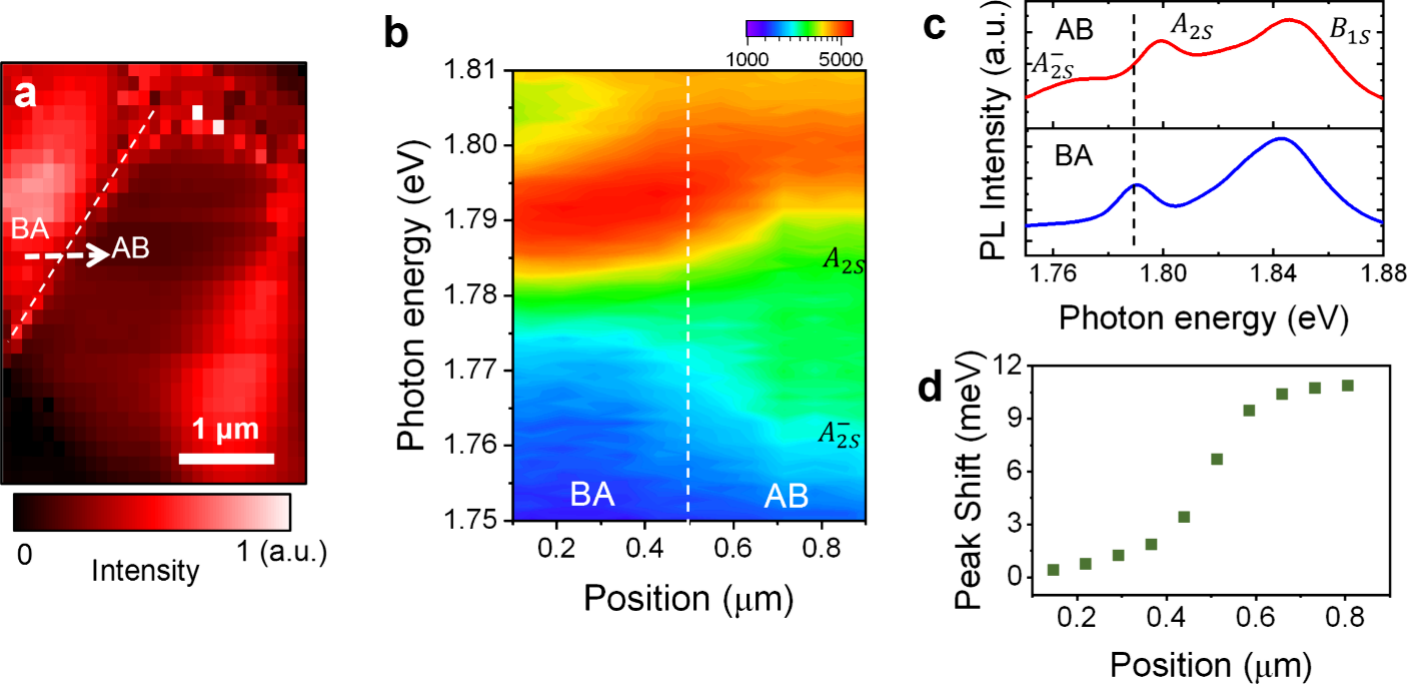
Rydberg exciton-polaron states. (a) Hyperspectral PL imaging with excitation CW laser of 2.33 eV at the same location as in Figure 3a. The measured temperature is 8 K. The intensity of the A_2S_ peak is plotted. (b) Contour plot of PL spectra along the white arrow in Figure 4(a). (c) Normalized PL spectra of A_2S_^–^, A_2S_, B_1S_ in AB and BA domain. The A_2S_ position shifted from 1.790 to 1.799 eV from the BA to the AB domain. (d) Peak shift as a function of position. Excited exciton-polaron states show a larger peak shift compared to the exciton-polaron.

Finally, we investigated the role of the moiré lattice size on the excitons in monolayer MoSe_2_ especially in smaller domain sizes (below 1 μm). KPFM images in Figure 5a shows four distinct moiré sizes on thBN, ranging from 111 to 252 nm. The corresponding PL spectra for both the repulsive polaron (A_1S_) and the attractive polaron (A_1S_^–^) are shown in Figure 5b,c, respectively. Due to the variation in the trion energy and initial electron density among different samples, the relative energy is plotted with respect to the moiréless region for each sample. Figure 5d quantifies this relationship, plotting the PL peak shift as a function of the inverse moiré size (1/λ). Notably, the RP (A_1S_) exhibits a consistent blue shift, while the AP (A_1S_^–^) displays a red shift, agreeing with previous observations.^24,25^ Additionally, the Fermi level change was estimated from the energy shift of the RP, as previously shown in Figure 3e,f. As shown in Figure 5e, we determined the strength of in-plane electric field as a function of moiré size using the maximum potential difference based on the moiré sizes in Figure 2d. This calculation was performed using the following equation: *E*_in-plane_ = (2π/λ)*V*_max_. As the moiré size decreases, the strength of the applied maximum electric field increases sharply, supporting the behavior we observe. The inset in Figure 5e schematically depicts this charge redistribution, highlighting the accumulation of electrons in specific domains due to the enhanced electric field for smaller moiré sizes.

**Figure 5:**
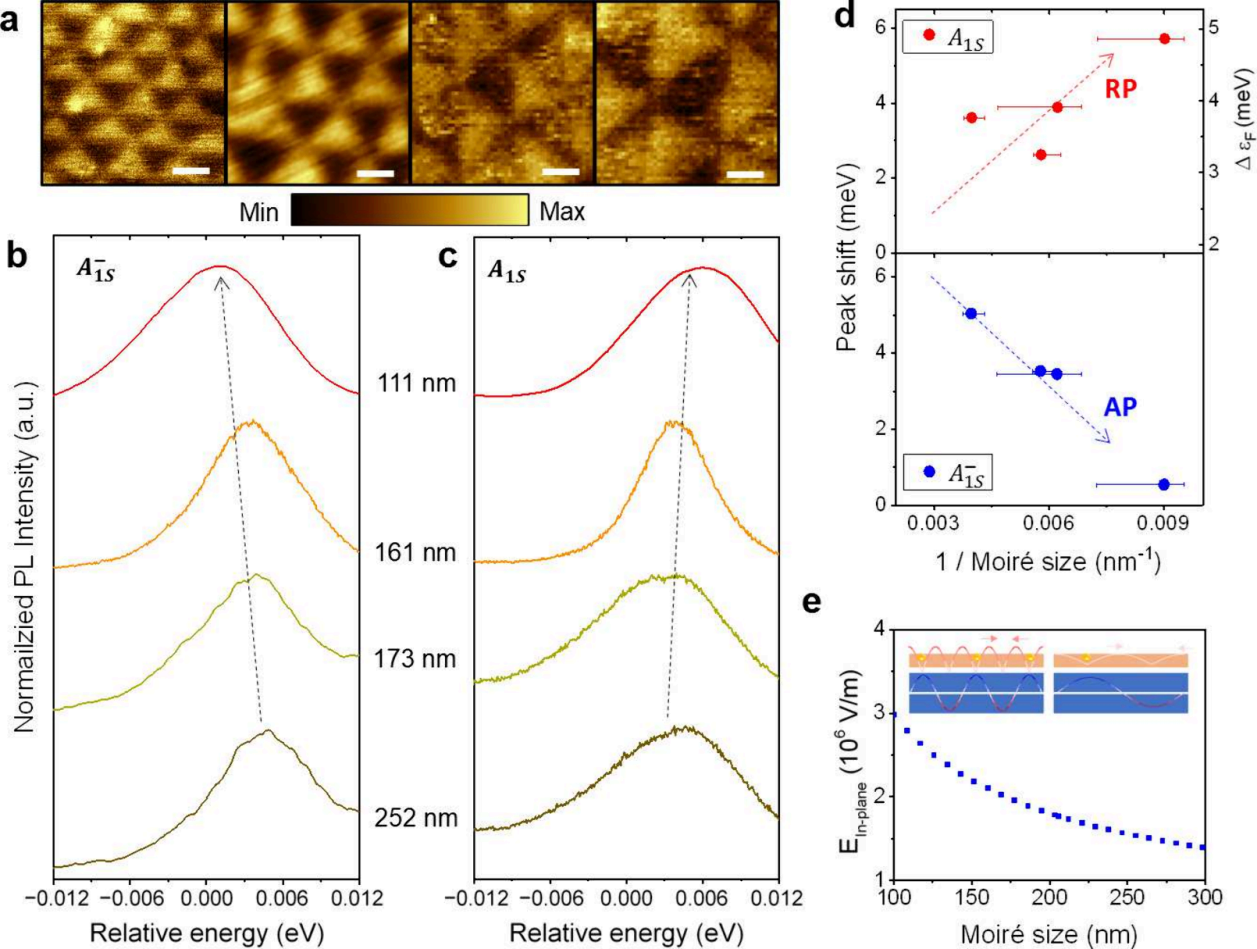
Exciton-polaron peak shift induced by moiré superlattice. (a) KPFM images on thBN regions where the PL of MoSe_2_ was measured. The moiré sizes, from left to right, are approximately 111 nm (top hBN 5 nm, bottom hBN 5 nm), 161 nm (top hBN 8.5 nm, bottom hBN 8.5 nm), 173 nm (top hBN 7 nm, bottom hBN 14 nm), and 252 nm (top hBN 7 nm, bottom hBN 14 nm). The scale bar is 100 nm. (b) Normalized A_1S_^–^ peak shift depending on the moiré size. (c) Normalized A_1S_ peak shift depending on the moiré size. The relative energy shift is plotted with respect to the moiré-less region for each sample. The excitation laser is a 2.33 eV CW laser, and the sample temperature is 8 K. (d) PL peak positions in Figure 4b,c as a function of 1/moiré size. The repulsive polaron exhibits a consistent blue shift, while the attractive polaron displays a red shift. The Fermi level change (Δϵ_F_) corresponds to the repulsive polaron peak shift. (e) Strength of the maximum in-plane electric field as a function of the moiré size, calculated from the potential differences shown in Figure 2d. The inset figure indicates that a large electric field is present at small moiré sizes.

In addition to the shift in exciton energies in the proximity of the moiré superlattice, another intriguing effect is the modification in the exciton–exciton interaction due to the moiré potential.^30,31^ Pump power dependent PL measurements show changes in saturation behavior of the PL of the excitons placed in the proximity of the moiré superlattice (Figure S3). The emission was found to saturate at lower pump power for the moiré proximitized repulsive polarons compared to the bare repulsive polarons. Interestingly, the attractive polarons did not show saturation behaviors. Further investigation of exciton–exciton interaction modified by the underlying moiré potential will be the subject of a future work. A more detailed analysis using Rydberg states, which can probe exciton behavior more sensitively as shown in Figure 4, or further studies with gate-controlled Fermi levels are required in the future.

In conclusion, we have demonstrated the imprinting of moiré patterns from twisted hBN onto monolayer MoSe_2_ through KPFM and PL measurements. We have proposed a method for creating small moiré angles below 0.01° by repeating the transfer process, allowing the realization of large domains, and observing the excitons and exciton-polaron behavior for the different domains through far-field spectroscopic measurements. The difference in Fermi levels between the domains results in distinct interactions between excitons and electrons, leading to domain-dependent energy shifts. The increased Fermi level in the AB domain induces the formation of exciton-polarons exclusively in this domain. Conversely, the excitons in the BA domain, where the Fermi level is reduced, approaches the neutral exciton state. Subsequently, we experimentally confirmed the optical property changes induced by the moiré pattern in monolayer MoSe_2_ by varying the moiré pattern size down to 111 nm, which is attributed to the charge redistribution by the in-plane electric field. These results highlight the potential of twisted hBN as a platform for controlling the optical and electronic properties of 2D materials for optoelectronic and valleytronic applications.

Note: During the finalizing of this manuscript, we became aware of two preprints that discuss confinement of trions^32^ and moiré metasurface^33^ using proximity effect similar to that reported here.

## Supporting Information Available

Details of the methods used for sample preparation, KPFM measurements, and hyperspectral imaging, schematic of the thBN sample fabrication process, additional topography, KPFM data of thBN following the first transfer process and the second transfer, hyperspectral PL map of MoSe_2_/thBN, pump power dependent PL and Raman spectrum of MoSe_2_, hyperspectral PL map of thBN/MoSe_2_/hBN, and line width of repulsive polaron and attractive polaron across the AB and BA domain (PDF)
