# Supplementary material for: Moiré Exciton Polaron Engineering via twisted hBN
Source: Nano Lett. 2024 Dec 2;25(4):1381–8. doi: 10.1021/acs.nanolett.4c04996 (PMC11783590; doi:10.1021/acs.nanolett.4c04996)
Supplement: Supplementary file 1 — nl4c04996_si_001.pdf [file nl4c04996_si_001.pdf]

# Supporting Information: Moiré exciton polaron engineering via twisted hBN

Minhyun Cho,[†,‡] Biswajit Datta,[‡] Kwanghee Han,[†] Saroj B. Chand,[¶] Pratap Chandra Adak,[‡] Sichao Yu,[‡] Fengping Li,[§] Kenji Watanabe,[‖] Takashi Taniguchi,[⊥] James Hone,[#] Jeil Jung,[§,@] Gabriele Grosso,[¶,△] Young Duck Kim,[*,†,∇] and Vinod M. Menon[*,‡]

†*Department of Physics, Kyung Hee University, Seoul 02447, Republic of Korea*

‡*Department of Physics, City College of New York, New York, NY 10031, United States*

¶*Photonics Initiative, Advanced Science Research Center, City University of New York, New York, NY, 10031, United States*

§*Department of Physics, University of Seoul, Seoul 02504, Republic of Korea*

‖*Research Center for Electronic and Optical Materials, National Institute for Materials Science, 1-1 Namiki, Tsukuba 305-0044, Japan*

⊥*Research Center for Materials Nanoarchitectonics, National Institute for Materials Science, 1-1 Namiki, Tsukuba 305-0044, Japan*

#*Department of Mechanical Engineering, Columbia University, New York, NY 10027, United States*

@*Department of Smart Cities, University of Seoul, Seoul 02504, Republic of Korea*

△*Physics Program, Graduate Center, City University of New York, New York, NY, 10016, United States*

∇*Department of Information Display, Kyung Hee University, Seoul 02447, Republic of Korea*

E-mail: ydk@khu.ac.kr; vmenon@ccny.cuny.edu

**Contents**

Methods

**1. Sample preparation**

**2. KPFM measurements**

**3. PL measurements and Hyperspectral imaging**

**Figure S1.** Schematic of the thBN sample fabrication process and repeating transfer process.

**Figure S2.** Additional topography, KPFM data of thBN in the first transfer process.

**Figure S3.** Hyperspectral of $MoSe_2$ / thBN, pump power dependent PL and raman spectrum of $MoSe_2$

**Figure S4.** Additional topography, KPFM data of thBN in the second transfer process of thBN.

**Figure S5.** Hyperspectral PL mapping results of thBN / $MoSe_2$ / hBN.

**Figure S6.** Line width of repulsive polaron and attractive polaron along the white arrow in Figure 3

**1. Sample preparation** thBN was fabricated by aligning the zigzag or armchair edge formed when the two hBNs are naturally exfoliated. hBN was exfoliated on a 90nm $SiO_2$/Si substrate using PC (Polycarbonate) dry transfer method. We exfoliated the flux-grown high quality $MoSe_2$ bulk crystal onto the PDMS (Polydimethylsiloxane) and transferred to hBN or thBN. After transfer process, samples were annealed at 300 °C for 1 hour under a nitrogen environment and contact cleaned[1] using AFM to remove strained regions, bubbles and the polymer residue.

**Fabrication technique for large domains**: To realize large domains, we repeated the process of pick and transfer. The technique of pick and transfer is similar to the one used in the first place as shown in the supporting Fig. S1. Owing to the tendency of hBN layers to relax to the 0 degree configuration, the repeated transfer process results in larger domains.

**2. KPFM measurement** We performed FM-KPFM (Frequency Modulated-Kelvin Probe Force Microscopy) measurement using Bruker's MultiMode 8 AFM. The tip used is PFQNE-AL, which has a frequency of 300 kHz and a spring constant of 0.8 N/m, and the tip radius is 5 nm. As it is commonly known, KPFM measures the work function of the sample which is an external potential that offsets the potential difference between the tip and the sample surface. Measurement methods are divided into AM-KPFM and FM-KPFM according to the feedback method. Among them, FM-KPFM has a better lateral resolution, making it easy to measure small moiré superlattices.[2] The Moiré potential can be measured by the difference in surface potential measured in AB and BA domains at hBN, and when positive bias is performed through the tip, the high potential area corresponds to the BA domain and the low potential area corresponds to the AB domain.[3,4]

**3. PL measurements and Hyperspectral imaging** We used home-built confocal microscope set up with continuous-wave (CW) green laser (532 nm) for excitation. An objective lens with NA = 0.9 allowed us to reach laser spot size of 1 µm$^2$. We used 42.9 µW

for the exciton states excitation and 386 µW for the Rydberg states. The set up is coupled with a EM-CCD for the high efficiency measurements. The sample is placed in a closed cycle cryostat (Montana) for the low temperature measurements. For the hyperspectral imaging, we used XY galvometer system in a 4f configuration.

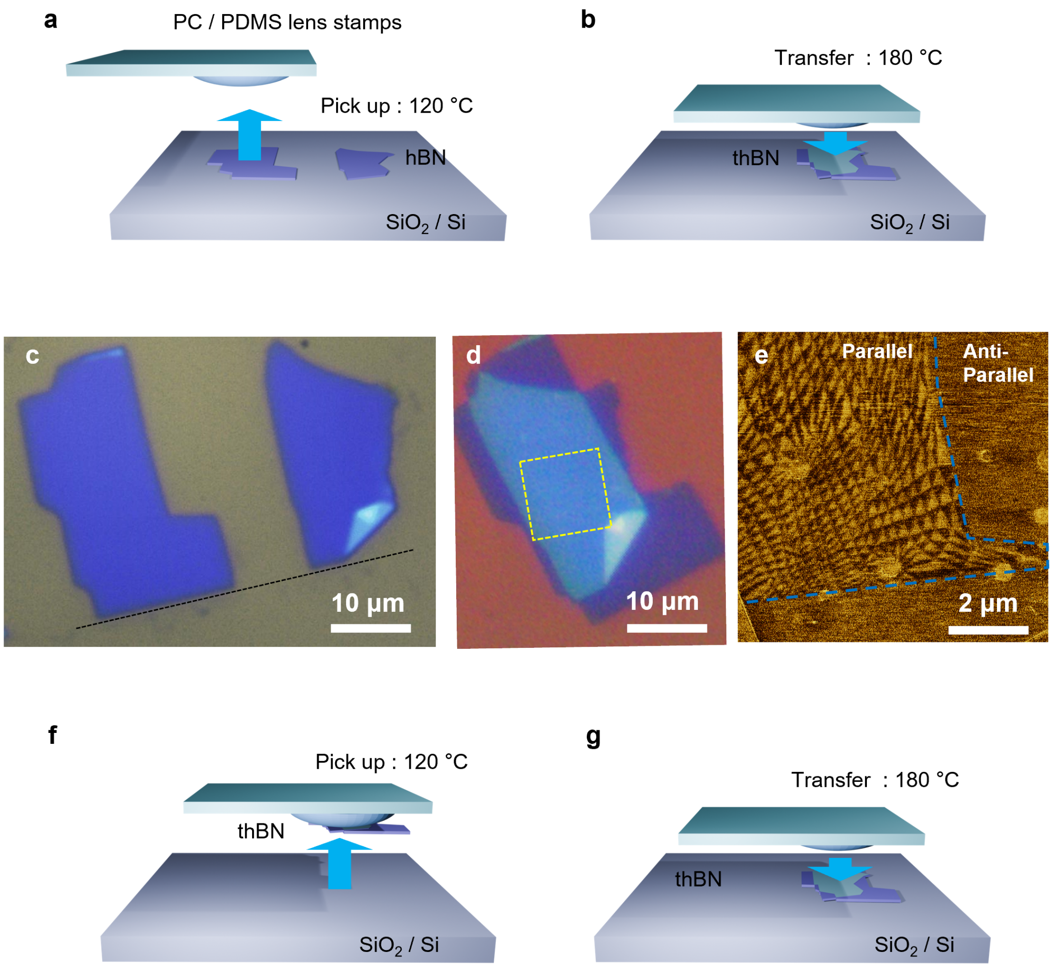


Figure S1: Schematic of the thBN sample fabrication process and repeating transfer process. (a), (b) First transfer process. The pick-up temperature is 120°C and the transfer temperature is 180°C to melt the PC film. The melted PC film is dissolved by soaking in chloroform for 24 hours. (c) Optical microscope image of two aligned hBN flakes in their as-exfoliated state. (d) Optical microscope Image of stacked thBN. (e) KPFM image of the yellow dotted area in (d). Monolayer steps along the blue dotted line show moiré patterns in the parallel stacking region. (f), (g) Second transfer process. Repeated under the same conditions as the first transfer process.

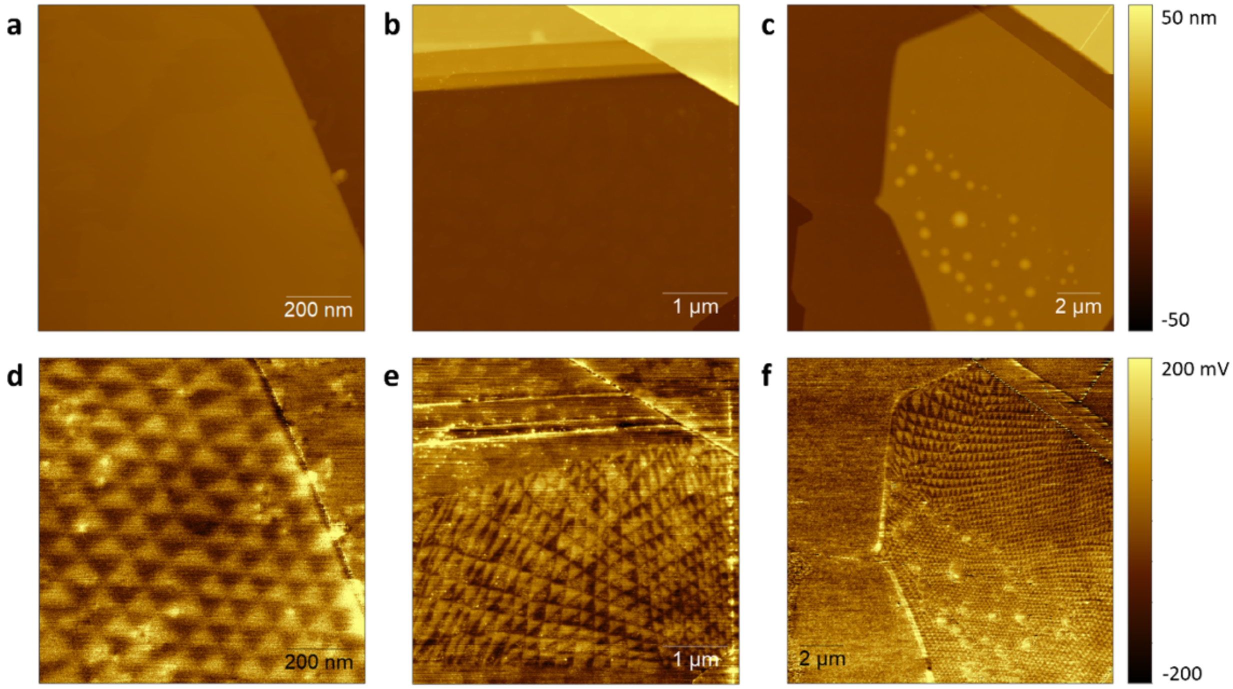


Figure S2: Additional topography, KPFM data of thBN in the first transfer process. We stacked the hBN flake aligned nearly perfectly in their as-exfoliated state. (a), (b), and (c) show the topography. The thickness of the top hBN 5 nm, 7 nm, 9 nm, and the bottom hBN is 5 nm, 14 nm, 16 nm. (d), (e), and (f) represent KPFM measurements, revealing moiré sizes and stacking angles: (d) moiré size of 111 nm and stacking angle of 0.129 degrees; (e) moiré sizes ranging from 173 nm to 658 nm, with corresponding stacking angles ranging from 0.0218 to 0.0829 degrees; and (f) moiré sizes ranging from 260 nm to 370 nm, with corresponding stacking angles ranging from 0.0388 to 0.0552 degrees.

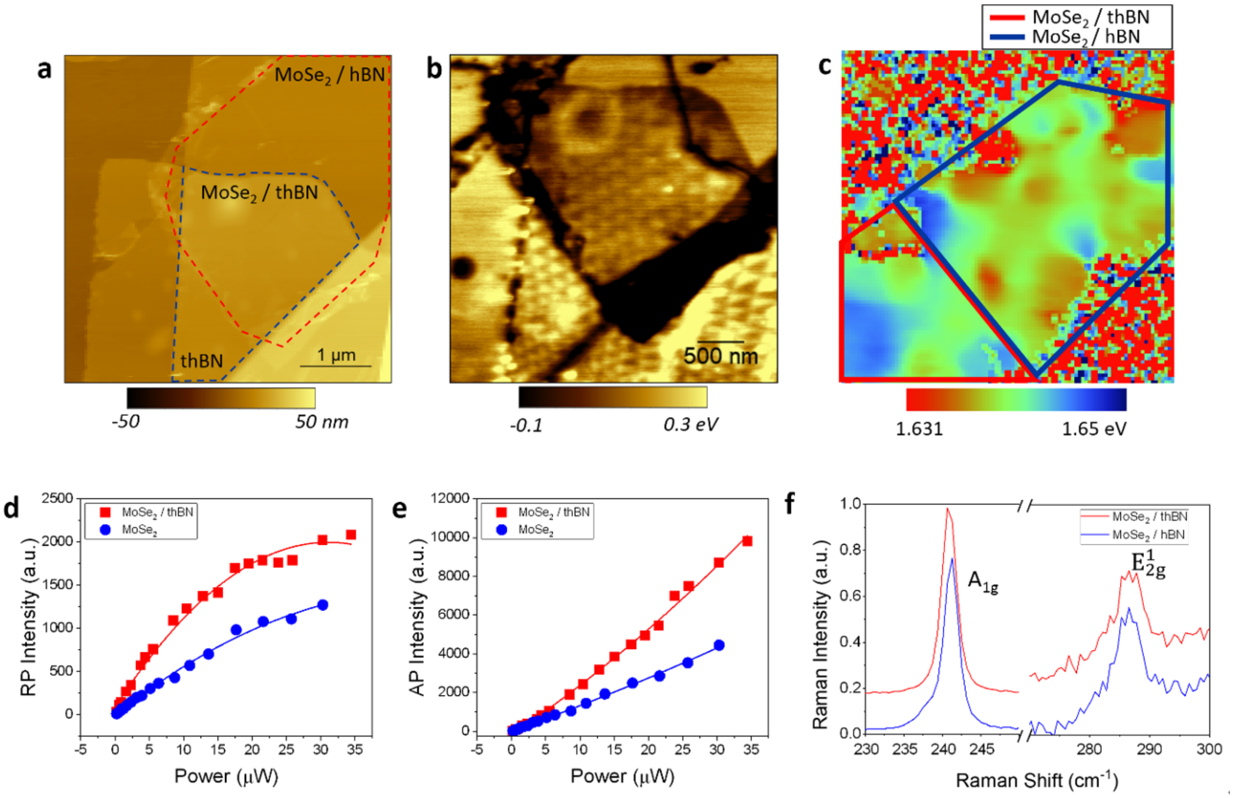


Figure S3: Hyperspectral of $MoSe_2$ / thBN, pump power dependent PL and raman spectrum of $MoSe_2$ (a) AFM topography image. $MoSe_2$ area is marked with a red dotted line. thBN area is marked with a blue dotted line. (b) KPFM image. The area above $MoSe_2$ has lower potential due to differences in work function, but the moiré pattern is identified the same. (c) Hyperspectra PL mapping image of repulsive polaron energy. Blue shift is observed in the thBN area. The power dependence of (d) repulsive polaron and (e) attractive polaron on the thBN or hBN. The solid line is the value fitted by the parabola function. (f) Raman spectrum of $MoSe_2$ on thBN and hBN.

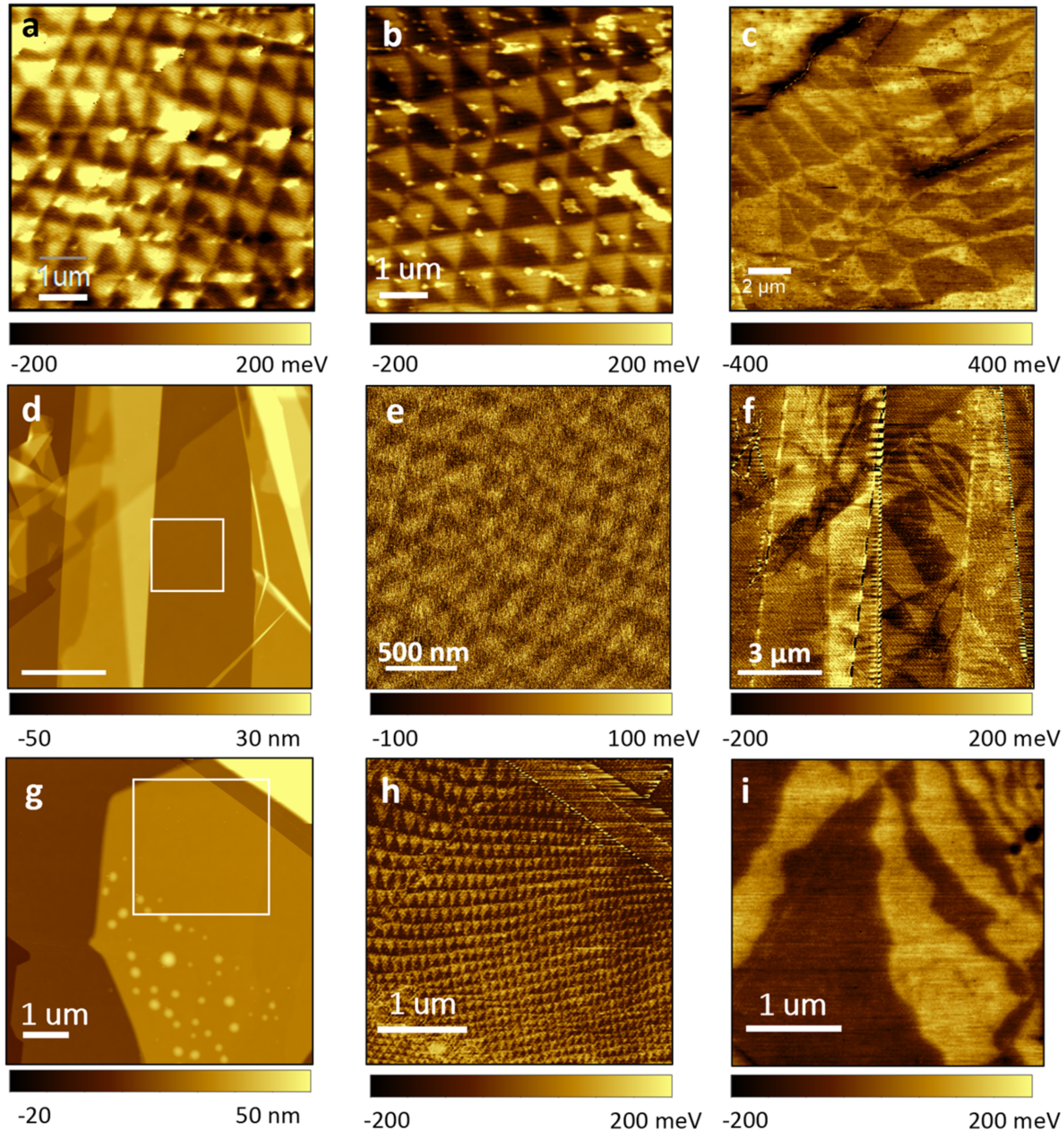


Figure S4: Additional topography, KPFM data of thBN in the second transfer process of thBN. (a) KPFM image immediately after stacking using the PC dry transfer method. (b) After annealing at 200 °C, 1h with 100 sccm nitrogen flow. We confirmed that the moire size does not change during the annealing process. (c) Moiré size is larger after second transfer process on the same $SiO_2$ / Si substrate. (d, g) Topography of the thBN. KPFM measured before (e, h) and after (f, i) the second transfer process on white box at (d, g).

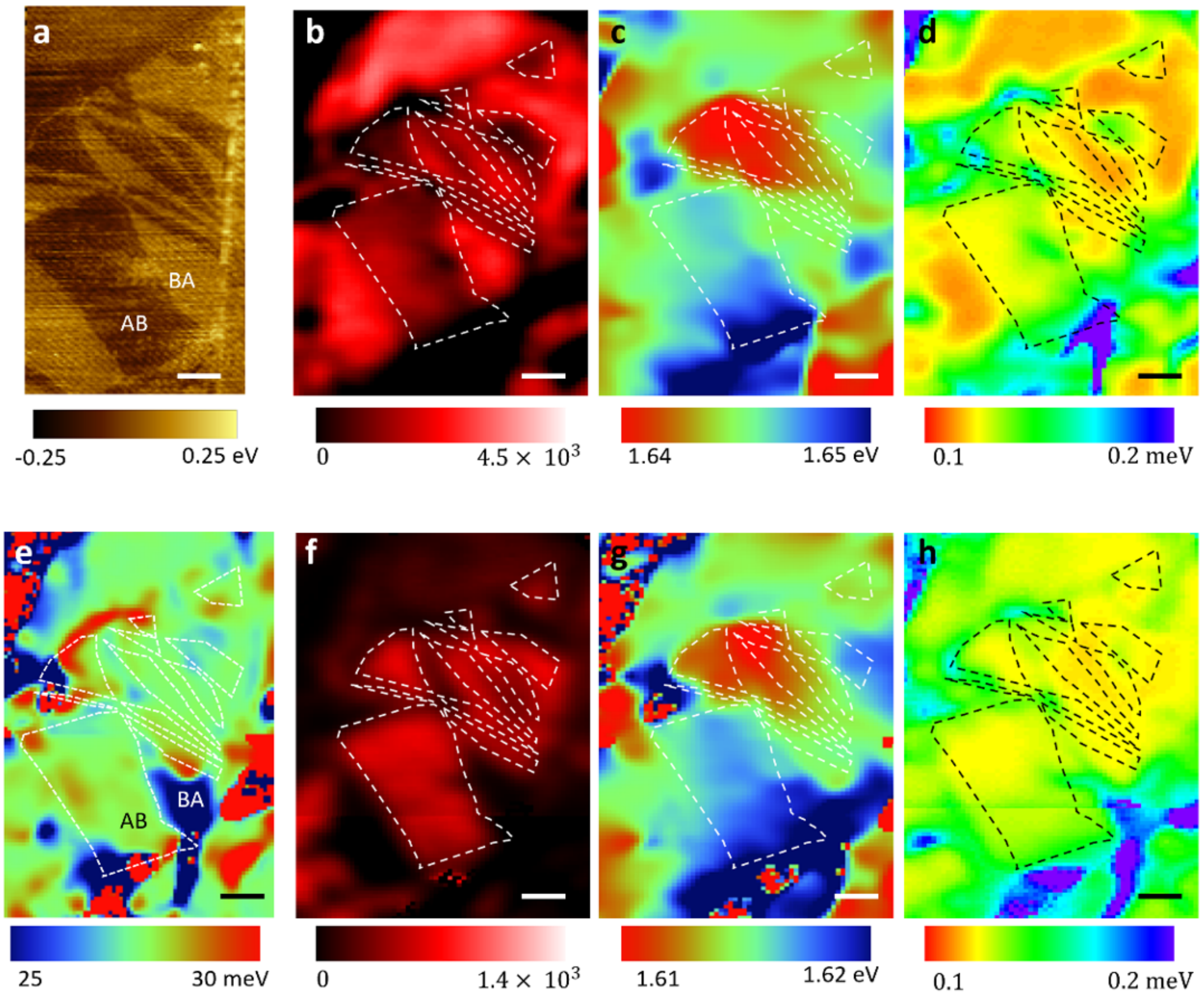


Figure S5: Hyperspectral PL mapping results of thBN / $MoSe_2$ / hBN. (a) KPFM image of sample. Repulsive polaron (b) intensity, (c) energy, (d) linewidth. (e) The difference between repulsive and attractive polaron energy. Attractive polaron (f) intensity, (g) energy, (h) linewidth. Scale bar is 1 µm.

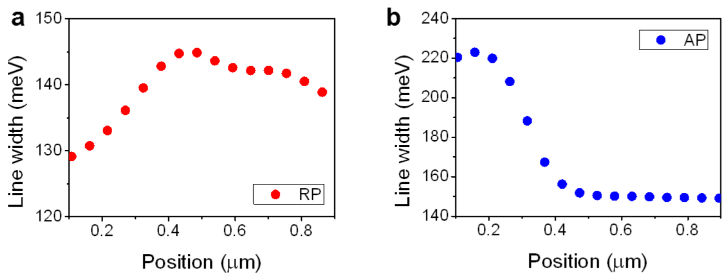


Figure S6: Line width of repulsive polaron and attractive polaron along the white arrow in Figure 3. (a) Repulsive polaron line width and (b) Attractive polaron line width.

# References


(1) Rosenberger, M. R.; Chuang, H.-J.; McCreary, K. M.; Hanbicki, A. T.; Sivaram, S. V.; Jonker, B. T. Nano-“squeegee” for the creation of clean 2D material interfaces. *ACS applied materials & interfaces* **2018**, *10*, 10379–10387.

(2) Zerweck, U.; Loppacher, C.; Otto, T.; Grafström, S.; Eng, L. M. Accuracy and resolution limits of Kelvin probe force microscopy. *Physical Review B* **2005**, *71*, 125424.

(3) Woods, C.; Ares, P.; Nevison-Andrews, H.; Holwill, M.; Fabregas, R.; Guinea, F.; Geim, A.; Novoselov, K.; Walet, N.; Fumagalli, L. Charge-polarized interfacial superlattices in marginally twisted hexagonal boron nitride. *Nature communications* **2021**, *12*, 347.

(4) Chiodini, S.; Kerfoot, J. R.; Venturi, G.; Mignuzzi, S.; Alexeev, E. M.; Rosa, B. T.; Tongay, S.; Taniguchi, T.; Watanabe, K.; Ferrari, A. C.; Ambrosio, A. Moiré Modulation of Van Der Waals Potential in Twisted Hexagonal Boron Nitride. *ACS nano* **2022**, *16*, 7589–7604.
